# Supplementary material for: Identification of Drug Repurposing Candidates for Coxsackievirus B3 Infection in iPSC-Derived Brain-like Endothelial Cells
Source: Int J Mol Sci. 2025 Jul 22;26(15):7041. doi: 10.3390/ijms26157041 (PMC12346471; doi:10.3390/ijms26157041)
Supplement: Supplementary file 1 [file ijms-26-07041-s001.zip › ijms-3750695-supplementary.pdf]

Jacob Wood, CVB Paper Table Draft

7/21/2025

Supplementary Tables:

Supplementary Table S1. Top 10 GSEA Down-Regulated Pathways 2 Days Post Infection

Pathway	p-Adj	Rank
Syncytium formation	<0.01	1
Cell-cell fusion	<0.01	2
Syncytium formation by plasma membrane fusion	<0.01	3
Hormone metabolic process	<0.01	4
Phenol compound metabolic process	<0.01	5
Hormone biosynthetic process	<0.01	6
Cellular modified AA metabolic process	<0.01	7
Cellular response to oxygen-containing compound	0.08	8
Response to oxygen-containing compound	0.08	9
Cellular response to nitrogen compound	0.09	10

Abbreviations: AA; Amino Acid

Supplementary Table S2. Top 10 GSEA Down-Regulated Pathways 5 Days Post Infection

Pathway	p-Adj	Rank
Cytoplasmic translation	<0.01	1
Cytosolic large ribosomal subunit	<0.01	2
Cytosolic ribosome	<0.01	3
Structural constituent of ribosome	<0.01	4
Cytosolic small ribosomal subunit	<0.01	5
Polysomal ribosome	<0.01	6
Ca-dependent adhesion via PM adhesion molecules	<0.01	7
Ribosomal subunit	<0.01	8
Catenin complex	<0.01	9
Small ribosomal subunit	<0.01	10

Abbreviations: PM; Plasma Membrane

Supplementary Table S3. Top 10 GSEA Leading-Edge Genes 2 Days Post Infection

Symbol	Name	N	Log2Fold Change
IL6	interleukin 6	162	2.10
TNF	tumor necrosis factor	151	1.81
CCL5	C-C motif chemokine ligand 5	116	1.35
ANXA1	annexin A1	104	0.36
MEF2C	myocyte enhancer factor 2C	103	0.46
NR4A3	nuclear receptor subfamily 4 group A member 3	84	3.07
ZC3H12A	zinc finger CCCH-type containing 12A	84	0.58
PTK2B	protein tyrosine kinase 2 beta	82	0.57
SNCA	synuclein alpha	82	0.47
CCL2	C-C motif chemokine ligand 2	79	0.64

Supplementary Table S4. Bottom 10 GSEA Leading-Edge Genes 2 Days Post Infection

Symbol	Name	N	Log2Fold Change
AGTR1	angiotensin II receptor type 1	15	-0.66
CGA	glycoprotein hormones, alpha polypeptide	14	-4.43
GCGR	glucagon receptor	14	-1.45
CRHR2	corticotropin releasing hormone receptor 2	14	-1.03
DRD4	dopamine receptor D4	14	-0.41
MYH6	myosin heavy chain 6	13	-0.88
CHRM1	cholinergic receptor muscarinic 1	13	-0.68
ROBO2	roundabout guidance receptor 2	13	-0.51
GNRHR2	gonadotropin releasing hormone receptor 2	12	-0.44
SULF1	sulfatase 1	12	-0.40

Supplementary Table S5. Top 10 GSEA Leading-Edge Genes 5 Days Post Infection

Symbol	Name	N	Log2Fold Change
TNF	tumor necrosis factor	275	5.37
IL6	interleukin 6	255	4.61
CCL5	C-C motif chemokine ligand 5	207	7.02
LGALS9	galectin 9	195	1.32
ZC3H12A	zinc finger CCCH-type containing 12A	162	1.17
CAV1	caveolin 1	149	1.28
GATA3	GATA binding protein 3	147	0.98
HLA-G	MHC, class I, G	146	3.16
CX3CL1	C-X3-C motif chemokine ligand 1	138	2.99
THBS1	thrombospondin 1	134	1.62

Abbreviations: MHC; Major Histocompatibility Complex

Supplementary Table S6. Bottom 10 GSEA Leading-Edge Genes 5 Days Post Infection

Symbol	Name	N	Log2Fold Change
SCN5A	Na voltage-gated channel alpha subunit 5	44	-0.98
KCNQ1	K voltage-gated channel subfamily Q	39	-0.79
WNT5A	Wnt family member 5A	39	-0.89
CACNA1C	Ca voltage-gated channel subunit a1 C	38	-0.6
CACNA1D	Ca voltage-gated channel subunit a1 D	36	-1.57
CACNA1G	Ca voltage-gated channel subunit a1 G	35	-2.01
CACNA2D1	Ca voltage-gated channel aux. subunit a2d 1	35	-0.72
LRRK2	leucine rich repeat kinase 2	34	-3.60
NRXN1	neurexin 1	32	-1.37
KCNH2	K voltage-gated channel (H) member 2	30	-0.67

Supplementary Table S7. Top 10 Up-regulated EnrichR Pathways 2 Days Post Infection

Pathway Name	p-Value	Rank
Cellular response to type i interferon	<0.01	1
Type i interferon signaling pathway	<0.01	2
Regulation of ribonuclease activity	0.03	3
Regulation of response to wounding	<0.01	4
Regulation of vascular wound healing	<0.01	5
Negative regulation of actin filament depolymerization	0.05	6
Negative regulation of BVEC proliferation involved in angiogenesis	0.05	7
Negative regulation of lens fiber cell differentiation	0.05	8
Regulation of nuclease activity	0.05	9
Regulation of type b pancreatic cell proliferation	0.05	10

Abbreviations: BVEC; Blood Vessel Endothelial Cell

Supplementary Table S8. Bottom 10 Down-regulated EnrichR Pathways 2 Days Post Infection

Pathway Name	p-Value	Rank
Retinal cone cell development	0.12	1
Retinal cone cell differentiation	0.12	2
Negative regulation of morphogenesis of an epithelium	0.12	3
Positive regulation of neurotransmitter secretion	0.12	4
Positive regulation of VEGF signaling pathway	0.12	5
Muscle myosin complex	0.01	6
Regulation of muscle system process	0.12	7
Dermatan sulfate metabolic process	0.12	8
Receptor transactivation	0.48	9
Cell migration involved in gastrulation	0.48	10

Supplementary Table S9. Top 10 EnrichR Up-regulated Pathways 5 Days Post Infection

Pathway Name	p-Value	Rank
Cellular response to type i interferon	<0.01	1
Type i interferon signaling pathway	<0.01	2
Antigen presentation of exogenous antigen via MHC I	<0.01	3
Antigen presentation of endogenous antigen via MHC I	<0.01	4
Antigen presentation of endogenous peptide antigen via mhc class i	<0.01	5
Regulation of nuclease activity	<0.01	6
MHC class i protein complex	<0.01	7
Negative regulation of viral process	<0.01	8
Interferon-gamma-mediated signaling pathway	<0.01	9
Negative regulation of viral genome replication	<0.01	10

Supplementary Table S10. Bottom 10 Down-regulated EnrichR Pathways 5 Days Post Infection

Pathway Name	p-Value	Rank
Heparan sulfate proteoglycan, enzymatic modification	0.05	1
Cellular response to K ion	<0.01	2
Response to K ion	<0.01	3
[Heparan sulfate]-glucosamine 3-sulfotransferase 1 activity	0.23	4
Kidney morphogenesis	0.22	5
Membrane depolarization during sa node cell action potential	0.22	6
Postsynaptic density protein 95 clustering	0.22	7
Regulation of vascular associated smooth muscle contraction	0.22	8
Voltage-gated Ca channel activity involved in cardiac muscle	0.41	9
Axon extension involved in axon guidance	0.13	10

Supplementary Table S11. Top 10 Concordant Perturbagens 2 Days Post Infection

Perturbagen	Concordance Score	Mechanism Of Action
MLS000718723	0.70	Unknown
Menadione	0.69	Mitochondrial DNA polymerase inhibitor
GNF-PF-159	0.69	Tyrosine phosphatase inhibitor
SMR000075319	0.69	Importin-beta transport receptor inhibitor
MLS000564806	0.68	Unknown
ST072947	0.68	Unknown
AC-1520	0.68	Unknown
KUC104255	0.67	Unknown
Bithionol	0.67	Autotaxin inhibitor
Gossypol	0.67	BCL inhibitor, MCL1 inhibitor

Supplementary Table S12. Top 10 Concordant Perturbagens 5 Days Post Infection

Perturbagen	Concordance Score	Mechanism Of Action
SB-218078	0.54	CHK inhibitor
Salermide	0.47	SIRT inhibitor
COL-3	0.47	Matrix metalloprotease inhibitor
MLS002703105	0.47	Unknown
Narciclasine	0.47	Coflilin, LIM, Rho-associated kinase activator
Suloctidil	0.46	Adrenergic receptor antagonist
Suloctidil	0.46	Adrenergic receptor antagonist
LSM-6189	0.46	Topoisomerase inhibitor
Wortmannin	0.46	PI3K inhibitor
AC1N9M1C	0.46	Unknown

Supplementary Table S13. Top 10 Concordant Perturbagen MOA 2 Days Post Infection

Mechanism of Action	Number of Perturbagens Associated
HDAC inhibitor	101
CDK inhibitor	93
PDGFR tyrosine kinase receptor inhibitor	90
VEGFR inhibitor	88
KIT inhibitor	82
PI3K inhibitor	72
FLT3 inhibitor	67
NFkB pathway inhibitor	64
Tubulin inhibitor	50
MTOR Inhibitor	40

Supplementary Table S14. Top 10 Discordant Perturbagen MOA 2 Days Post Infection

Mechanism of Action	Number of Perturbagens Associated
VEGFR inhibitor	20
MEK inhibitor	18
PTK ABL inhibitor	17
Discoidin domain receptor 2 inhibitors	16
FGFR 1 inhibitor	16
FGFR 2 inhibitor	16
MAP kinase p38 beta inhibitor	16
PDGFR inhibitor	16
STK/PTK kinase B-raf inhibitor	16
STK/PTK kinase raf inhibitor	16

Supplementary Table S15. Top 10 Concordant Perturbagen MOA 5 Days Post Infection

Mechanism of Action	Number of Perturbagens Associated
CDK inhibitor	20
Cell cycle inhibitor	20
Peptidase inhibitor	19
ATPase inhibitor	17
Tubulin inhibitor	14
MTOR inhibitor	13
Topoisomerase inhibitor	10
PI3K class I inhibitor	9
STK MTOR inhibitor	9
NFkB pathway inhibitor	8

Supplementary Table S16. Top 10 Discordant Perturbagen MOA 5 Days Post Infection

Mechanism of Action	Number of Perturbagens Associated
PI3K inhibitor	30
PDGFR PTK inhibitor	24
BCR-ABL inhibitor	22
HDAC inhibitor	22
SRC inhibitor	21
PTK inhibitor	17
EGFR inhibitor	16
Ephrin receptor inhibitor	16
KIT inhibitor	15
Tyrosine-protein kinase BRK inhibitor	12
